# Supplementary material for: New Insights into MdSPS4-Mediated Sucrose Accumulation under Different Nitrogen Levels Revealed by Physiological and Transcriptomic Analysis
Source: Int J Mol Sci. 2022 Dec 16;23(24):16073. doi: 10.3390/ijms232416073 (PMC9782777; doi:10.3390/ijms232416073)
Supplement: Supplementary file 1 [file ijms-23-16073-s001.zip › Table S1.pdf]

Table S1 qRT-PCR primers for expression analysis of sugar and acid related genes in apple

Gene	（5′–3′）Forward primer	（5′–3′）Reverse primer
MdGAPDH	TCGAAGGATTGGAGAGGTGGAAGG	CGACGGTAGGAACACGGAATGC
MdENO1	GTTCAGGCTCCGTCCAATGCTC	CACCGACTTGACCTTGTACTCCTTG
MdFBA2-1	GATACTCTGCTCATCCGTGCCAAG	GCCTCCTCAGACTCTCCTTCTCC
MdFBA2-2	AATGGCGTACCGTTGTGAGCATC	TGTCTTGAGCAATGGAAGCGTAGC
MdHXK1	CCTTGCTGCTGCTGGAGTCTTG	CCACCATCCAAAGCTACCACTGTC
MdHXK4	ATCAAACGCCACAAACCACATAAGC	CCACCTTCCCCATACCCCTGAC
MdSPS2	CCTCCTGGCATGGACTTCAGTAATG	AACCGCATCACTTCTGACCATATCG
MdSPS3	GTCTCGTCCTGACCCCAAGA	AGTTGGCTAGCTCCCGAAGA
MdSPS4	GTGTGAAGCCAGGAGCCAAGAC	AGTGTAGACAAGGTTGCATCGGAAG
MdSS2	CAGAGGCAATTCCGTGGATGATGG	AGCATCTCTTCCAGGTCAGGTCTC
MdAGPS1	CCTCAATCGTCACCTTGCTCGTAC	ACCATCTCTTCCCAGCGTCTCC
MdAGPS2	CATTAACCAAGAAGCGTGCGAAGC	GATTGAGCGAAGCGGAATTGAACTG
MdPK2-1	TGAGGTATCCGACATTGCCATTGC	CAGTCCGAAGTGCGACAGTGTG
MdPK2-2	CCCAGTCACAATGTCGCAGTCC	ATTGAAGGTGGAGAGGAAGGAGGAG
MdDLD2	TCGGATGCGTGAGCTTCAGAATG	AGCCACTCCCTGTCTGTCATATCC
MdDLAT5-1	TTGATGCTATTCTGCCACCTGGAAC	CGACCATCCTTAGTAGCCACAACAG
MdDLAT5-2	GCTATTCTGCCGCCTGGAACTG	GTTCTTCCGTCCAATCCGACCATC
MdPPC16-1	CCAGAATCAGTTGTCCGCAGAGTG	GTATTCCACCAGCTTGTCGTCCTC
MdPPC16-2	CGTGTGATTCTCGGTGATGTAAGGG	CAGTGTTGCCTCCTCTGGAATGTC
MdMDH2	AAAGGTGTGGATGTGGTGGTCATTC	CACAGCCTCGATCAAGTTCCTCAC
GAPDH	TTCTCGTTGAGGGCTATTCCA	CCACAGACTTCATCGGTGACA
1300-MdSPS4	GAGCTCGGTACCCGGGGATCCTATGGCGGGAAACGACTG	CATGTCGACTCTAGAGGATCCAAATCCCTTGATCCCACGTG
TRV-MdSPS4	AGAAGGCCTCCATGGGGATCCATGGCGGGAAACGACTGG	CGTGAGCTCGGTACCGGATCCAAATCCCTTGATCCCACGTGC
