# Supplementary figures and images for: New Insights into MdSPS4-Mediated Sucrose Accumulation under Different Nitrogen Levels Revealed by Physiological and Transcriptomic Analysis
Source: Int J Mol Sci. 2022 Dec 16;23(24):16073. doi: 10.3390/ijms232416073 (PMC9782777; doi:10.3390/ijms232416073)

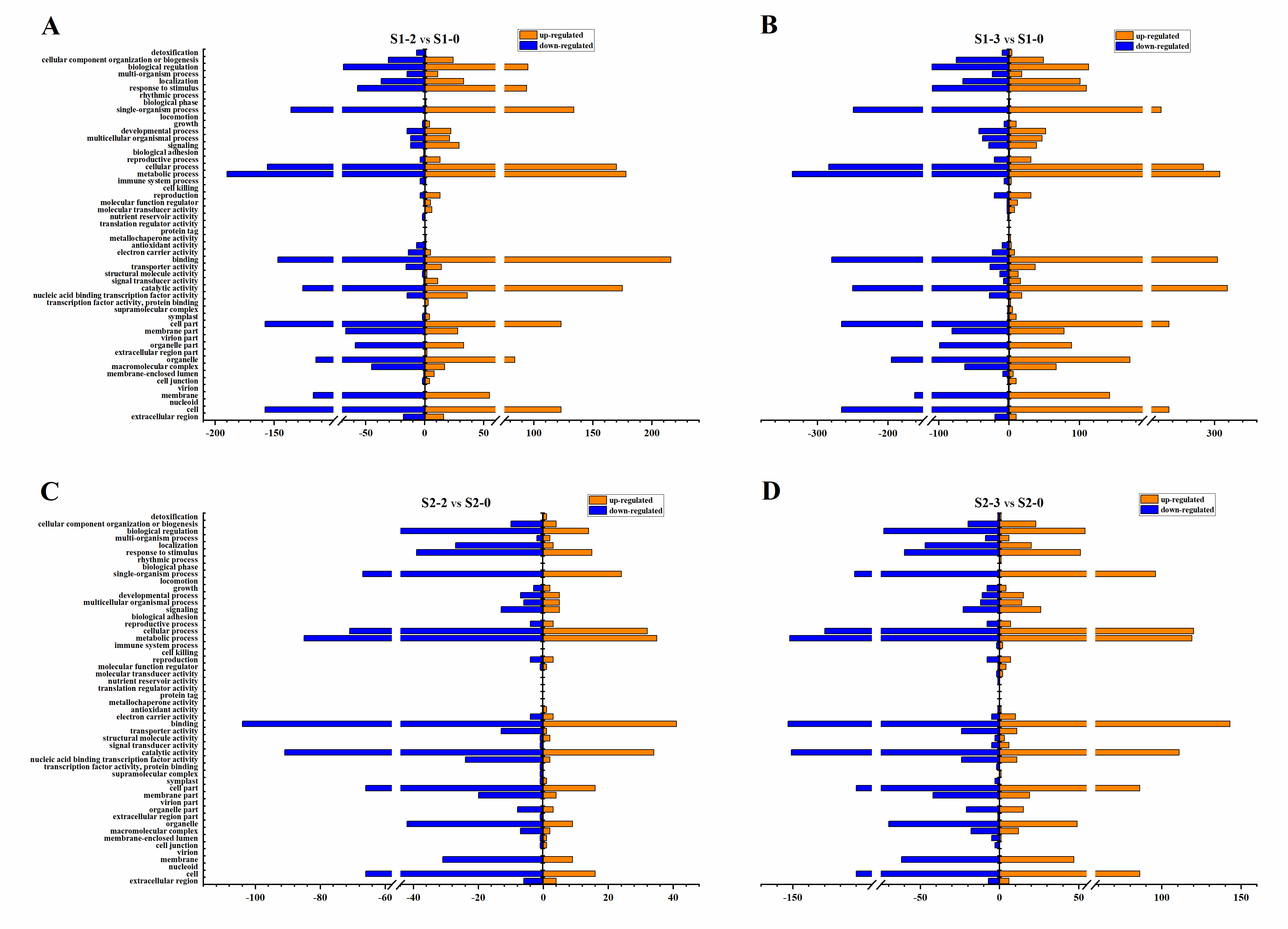

Supplement: Supplementary file 1 [file ijms-23-16073-s001.zip › Figure S1.tif]

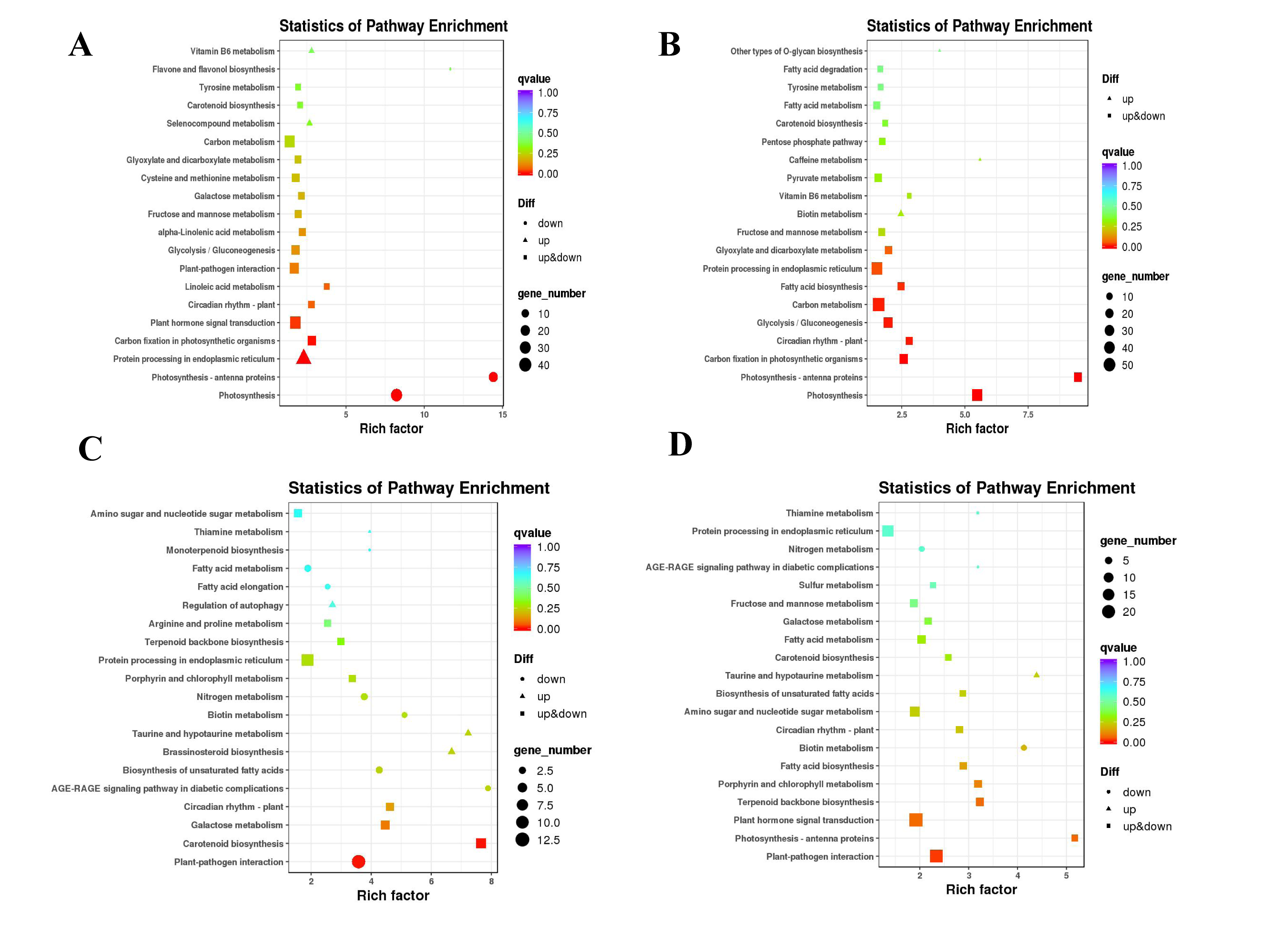

Supplement: Supplementary file 1 [file ijms-23-16073-s001.zip › Figure S2.tif]

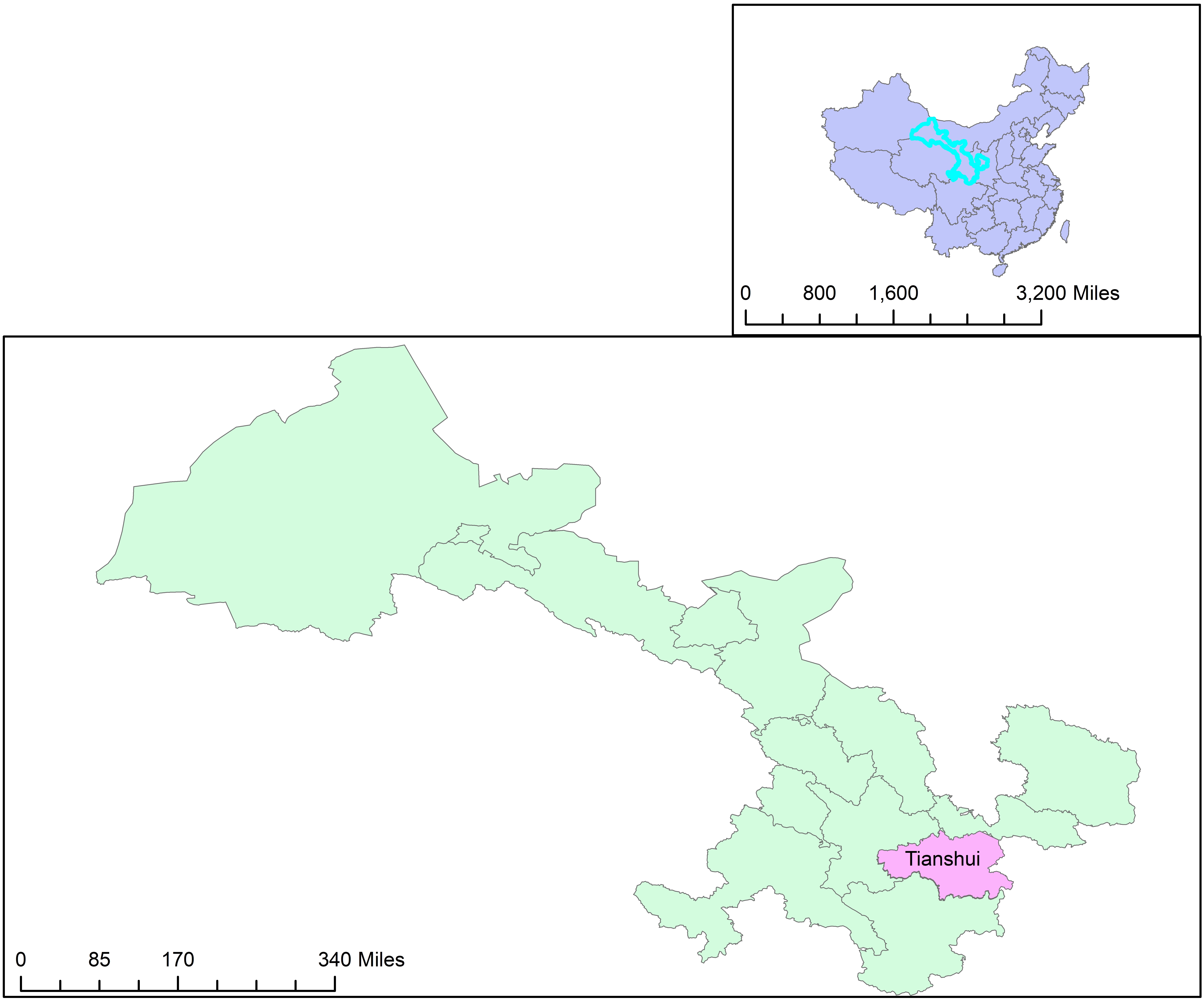

Supplement: Supplementary file 1 [file ijms-23-16073-s001.zip › Figure S3.jpg]
